# A mixed-methods approach to the psychological predictors of boredom in second language learning: mindfulness, grit, and self-regulation

**DOI:** 10.3389/fpsyg.2025.1609330

**Published:** 2025-08-22

**Authors:** Jingjing Lyu

**Affiliations:** School of Foreign Languages, Yulin Normal University, Yulin, China

**Keywords:** mindfulness, grit, self-regulation, L2 boredom, English language learning, mixed methods, Chinese EFL learners

## Abstract

This mixed methods study explores the relationships among mindfulness, grit, self-regulation, and L2 boredom in Chinese undergraduate English majors. Using structural equation modeling (SEM) with a sample of 516 students from various universities, the quantitative phase found that mindfulness and grit were negatively related to L2 boredom, with self-regulation partially mediating these relationships. Mindfulness and self-regulation were the strongest predictors of reduced boredom, while grit had a smaller yet significant impact. Multi-group analysis showed these relationships were consistent across gender and years of English learning experience. The qualitative phase, involving focus group discussions with 40 participants, offered insights into students’ experiences. Boredom was described as a complex emotional, cognitive, and behavioral state leading to disengagement. Mindfulness helped students maintain focus, grit provided perseverance, and self-regulation offered strategies to manage boredom. Additionally, dynamic teaching methods and supportive environments were identified as crucial for reducing boredom and enhancing engagement. These findings enhance the understanding of psychological factors influencing L2 boredom and suggest practical strategies for educators to foster mindfulness, grit, and self-regulation, thereby improving student engagement and learning outcomes.

## 1 Introduction

Psychological factors play a vital role in second language (L2) acquisition, a key focus in educational psychology (Dörnyei et al., 2015). While cognitive factors have long been prioritized, affective and motivational variables are increasingly seen as critical to language learning outcomes (Dörnyei, 2009; Lamb, 2017; Oxford, 2016). Mindfulness, grit, and self-regulation stand out as important constructs. While research is beginning to explore their individual connections to negative emotions like L2 boredom (e.g., Fathi et al., 2023; Solhi et al., 2023), a comprehensive understanding of how these factors might interact and collectively influence L2 learning experiences warrants further attention.

Boredom, characterized by disinterest and mental disengagement, is a pervasive challenge in education with significant negative effects (Sharp et al., 2017). In second language (L2) acquisition, it particularly impairs cognitive processing and hinders effective language learning (Li, 2022; Li et al., 2023). Despite its considerable impact, boredom has historically received less attention than more widely studied emotions like anxiety and motivation (Pawlak et al., 2020; Tze et al., 2016). This underscores an urgent need for continued examination of its predictors and mitigators specifically within L2 learning contexts.

To address this critical gap, this study investigates three key psychological predictors of L2 boredom: mindfulness, grit, and self-regulation. Each of these constructs offers a unique lens through which to understand and potentially mitigate disengagement. Mindfulness, the practice of non-judgmental present-moment awareness, is known to enhance cognitive function, reduce anxiety, and boost engagement (Kabat-Zinn, 2015; Khoury et al., 2013; Shapiro et al., 2006). Grit, defined as perseverance and passion for long-term goals, consistently supports academic success and persistence in demanding endeavors like language learning (Duckworth et al., 2007; Duckworth and Quinn, 2009; Eskreis-Winkler et al., 2014; Teimouri et al., 2022). Finally, self-regulation, the active process of managing one’s own learning experiences, reliably predicts positive educational outcomes and effective strategy use (Boekaerts and Cascallar, 2006; Greene, 2017; Schunk and Zimmerman, 2023; Zimmerman, 2002).

Although emerging research investigates individual or paired links between mindfulness, grit, self-regulation, and L2 boredom (e.g., Cheng, 2023; Mohammad Hosseini et al., 2023; Pawlak et al., 2022), few studies have examined these three predictors simultaneously to understand their combined influence and relative contributions to L2 boredom. Furthermore, how self-regulation might mediate the relationships of both mindfulness and grit with L2 boredom, particularly when examined concurrently within a single model, remains unclear. Additionally, there is a lack of in-depth, qualitative understanding of how students perceive and utilize these psychological resources in combination when facing boredom in their language classes. This study addresses these specific gaps using a mixed methods approach, integrating quantitative analysis to model the combined effects and mediation pathways with qualitative insights into students’ lived experiences.

This mixed methods research offers a detailed view of these relationships. The quantitative phase tests statistical links, while the qualitative phase explores students’ perceptions and strategies related to mindfulness, grit, self-regulation, and L2 boredom. The study addresses three questions: (1) What are the relative direct effects of mindfulness, grit, and self-regulation on L2 boredom? (2) Does self-regulation mediate the relationships between mindfulness and grit with L2 boredom when considered together? (3) How do students experience the interplay of these constructs in English language learning?

## 2 Literature review

### 2.1 Boredom in language learning

Boredom is a notable challenge in educational settings, marked by a lack of interest, mental disengagement, and feelings of monotony (Sharp et al., 2017). Although common, it has received less attention in educational research compared to emotions like anxiety, motivation, and enjoyment (Pawlak et al., 2020; Tze et al., 2016). Its effects are significant, including reduced motivation, lower academic performance, and higher dropout rates (Eastwood et al., 2012; Pekrun et al., 2010).

In L2 learning, boredom is often understood through its roots in learners’ perceptions. For instance, Control-Value Theory (Pekrun, 2006) suggests boredom emerges when students perceive a lack of control over their learning or find tasks lacking value, which leads to emotional disengagement. This perspective highlights boredom’s detrimental role in L2 learning, as it can undermine instructional effectiveness and impede language acquisition by blocking effective processing of new input (Krashen, 1982). Indeed, in L2 classrooms, the impact of boredom is particularly strong due to the sustained engagement language learning requires (Li et al., 2023). It hampers students’ ability to process and retain linguistic input (Nakamura et al., 2021), reducing their willingness to engage in practice. This limits opportunities for interaction and slows language development (Chen et al., 2022; Derakhshan and Fathi, 2025). Research further shows boredom predicts lower L2 achievement (Li and Wei, 2023) and leads to maladaptive coping strategies, such as avoidance, which obstruct progress (Solhi et al., 2023).

Several factors contribute to L2 boredom, reflecting an interplay of learner-internal and external elements. Key triggers include task characteristics like difficulty, lack of comprehension, or a mismatch with learner abilities (Nakamura et al., 2021; Pawlak et al., 2020). Both overly simple and excessively complex tasks can foster disengagement (Chapman, 2013; Li and Han, 2024).

The consequences of boredom extend beyond disengagement. It impairs cognitive processing, reducing attention to linguistic input and hindering retention of vocabulary and grammar (Chapman, 2013). Students experiencing frequent boredom may adopt avoidance behaviors, creating a cycle of disengagement that worsens learning challenges (Solhi et al., 2023). Addressing these consequences requires a multi-faceted approach that considers both student disposition and pedagogical strategies. To address this, educators need to design lessons that are engaging and appropriately challenging, incorporating diverse activities, student choice, and scaffolding to sustain interest (Zhang et al., 2022). A classroom environment that promotes active participation and values student contributions can also help reduce monotony and enhance the learning experience.

### 2.2 Grit in language learning

Grit, defined as perseverance and passion for long-term goals (Duckworth et al., 2007), is a key focus in educational research. Rooted in positive psychology, it emphasizes sustained effort and interest despite challenges (Credé, 2018; Duckworth et al., 2007; Duckworth and Quinn, 2009). Duckworth et al. (2007) identify two dimensions: perseverance of effort, reflecting commitment to tasks amid difficulties, and consistency of interest, indicating focus on goals despite distractions (Credé et al., 2017; Datu et al., 2017). Grit predicts academic success beyond traditional measures like IQ, as gritty individuals engage in deliberate practice essential for skill mastery (Duckworth and Quinn, 2009; Ericsson et al., 1993; Eskreis-Winkler et al., 2014). This sustained dedication is critical in education, where long-term effort drives complex skill development.

In language learning, which demands prolonged effort and practice, grit is highly relevant (Teimouri et al., 2022). Learners with greater grit persist through difficulties, improving outcomes (Fathi et al., 2024; Wei et al., 2020). L2 grit adapts the general concept to language-specific challenges, capturing the perseverance and passion needed for proficiency (Demir, 2024; Teimouri et al., 2021; Teimouri et al., 2022). Research shows L2 grit predicts important outcomes, including language achievement (even in online contexts, often indirectly through positive emotions like enjoyment; Zhao et al., 2024) and vocabulary acquisition (Alamer, 2021). It also positively relates to key motivational variables such as motivational intensity (Sun et al., 2024) and willingness to communicate (WTC; Lee, 2022; Sun et al., 2024). Grittier learners tend to practice consistently, overcome obstacles, and reach higher proficiency (Teimouri et al., 2022).

L2 grit interacts with other psychological factors. For instance, it can moderate the link between anxiety and achievement (Zhao and Wang, 2023). It also supports effective boredom coping strategies (Solhi et al., 2023). Research increasingly suggests L2 grit relates negatively to boredom across different learning contexts, including online Chinese language learning (Zhao and Wang, 2024), likely because grittier students persevere through monotonous tasks and sustain progress (Pawlak et al., 2022; Zhao and Wang, 2023). Notably, L2 grit has been found to play a mediating role in the relationship between perceived foreign language teacher support and foreign language anxiety, suggesting that supportive teaching can foster grit, which in turn reduces anxiety (Liu et al., 2025b). Moreover, studies exploring diverse profiles of L2 grit, such as “great effort and interest,” reveal how different configurations of perseverance and passion can uniquely influence the relationship between foreign language anxiety and achievement (Liu et al., 2025a). Interestingly, recent studies suggest the “perseverance of effort” (PE) facet of L2 grit may be particularly crucial in predicting performance (e.g., speaking performance; Sun et al., 2024) and managing negative emotions in challenging contexts like online learning (Zhao and Wang, 2024), while the “consistency of interest” (CI) facet might exert its influence more indirectly on achievement through variables like WTC (Sun et al., 2024) or positive emotions (Zhao et al., 2024). The role of grit appears prominent in autonomous settings as well, fostering goal adherence despite challenges (Alamer, 2022; Jin, 2024).

### 2.3 Self-regulation in language learning

Self-regulation is central to effective learning, involving processes where learners manage their educational experiences through cognitive, metacognitive, and motivational strategies. It includes setting goals, monitoring progress, self-assessing, and adjusting behaviors (Schunk and Zimmerman, 2023; Zimmerman, 2000). In academic settings, self-regulation is linked to higher achievement, persistence, and effective strategy use (Boekaerts and Cascallar, 2006; Greene, 2017; Zimmerman, 2002). Its role is especially critical in L2 acquisition, where learners face complex linguistic, cultural, and task-related challenges over time (Oxford, 2016; Rose et al., 2018; Seker, 2016).

Theoretical models provide insight into self-regulated learning. Zimmerman’s (2000) cyclical model, with forethought, performance, and self-reflection phases, underscores its dynamic nature. Pintrich (2000) integrates cognitive and motivational elements, highlighting metacognition, self-efficacy, and goal orientation. Bandura’s (1986) social cognitive theory emphasizes self-efficacy as a driver of self-regulatory behaviors. Applied to L2 learning, these models show how learners set language goals, choose strategies, track progress, and adapt to improve proficiency (Tsuda and Nakata, 2013), contributing to success in navigating linguistic and cultural complexities (Oxford, 2016; Rose et al., 2018; Seker, 2016).

Research confirms self-regulation’s positive impact on L2 outcomes (Derakhshan and Fathi, 2024). Self-regulated learners display greater motivation, better comprehension and retention, and adaptability to tasks (Sasaki et al., 2018). They use techniques like goal setting, organizing study time, seeking feedback, and applying metacognitive skills to refine their learning (Seker, 2016). Studies show self-regulation boosts L2 proficiency, with these learners outperforming peers in tests and communication (Teng and Zhang, 2022). It also mediates the link between motivation and performance, enhancing proficiency through self-efficacy and intrinsic motivation (Nitta and Baba, 2015; Zimmerman and Kitsantas, 2014).

Investigations reveal specific self-regulation strategies aiding L2 success. Oxford (2016) notes that self-regulated learners excel at tailoring strategies to their needs and styles. Teng and Zhang (2022) demonstrate that self-regulation skills transfer across domains, speeding L2 progress. Research with Japanese EFL students shows that goal setting, planning, and self-monitoring improve proficiency and satisfaction (Tsuda and Nakata, 2013). Beyond individual traits, the learning environment matters. Supportive settings fostering autonomy, feedback, and planning enhance self-regulation (Torres, 2013), as do digital tools for self-monitoring and assessment (Sasaki et al., 2018).

In conclusion, self-regulation is vital for L2 learning, enabling learners to control their progress, achieve goals, and adapt to challenges. Theoretical models clarify its cognitive, metacognitive, and motivational components, while research highlights its role in enhancing outcomes. Promoting self-regulated strategies and supportive environments helps learners become autonomous, motivated, and proficient in their target language. Building on these frameworks, particularly the cyclical nature of self-regulation involving planning, monitoring, and reflection (Zimmerman, 2000) and the importance of self-efficacy beliefs (Bandura, 1986), this study conceptualizes self-regulation as a potential mechanism. It is hypothesized that the capacity for effective self-regulation allows learners to translate dispositional traits like mindfulness and grit into specific actions—such as employing appropriate learning strategies, maintaining focus, and managing effort—that directly counteract feelings of boredom during language study.

### 2.4 Mindfulness

Mindfulness, defined as present-moment awareness with a non-judgmental attitude, is increasingly recognized in educational research for improving learning and well-being (Kabat-Zinn, 2015; Khoury et al., 2013; Shapiro et al., 2006). Originating from Buddhist traditions and adapted by Kabat-Zinn (2003), it is integrated into interventions targeting cognitive and psychological outcomes (Kabat-Zinn, 2003). In education, mindfulness enhances academic performance, emotional regulation, and student well-being (Fathi et al., 2023; Zeilhofer and Sasao, 2022).

Research highlights mindfulness’s impact on cognitive functions vital for learning, such as attention, memory, and executive control. It improves these by sharpening focus and reducing cognitive load (Zeilhofer and Sasao, 2022). Zeilhofer (2023) found that college students practicing mindfulness showed better short-term vocabulary retention in foreign language contexts, indicating its role in boosting focus and efficiency. Mindfulness also supports academic performance through emotional regulation, helping students manage stress and anxiety that hinder cognition (Ghanizadeh et al., 2019; Morgan and Katz, 2021). Students practicing mindfulness handle stressors effectively, maintaining clarity and improving performance on tasks requiring sustained attention (Wu and Zhao, 2023).

Mindfulness-Based Interventions (MBIs), like Kabat-Zinn’s (2003) Mindfulness-Based Stress Reduction (MBSR), reduce stress and anxiety, enhancing academic outcomes (Kabat-Zinn, 2003; Morgan and Katz, 2021). Morgan and Katz (2021) showed in a randomized trial that students in a mindfulness program had lower foreign language anxiety and greater language skill gains than controls, underscoring MBIs’ value in language education.

In EFL settings, mindfulness boosts academic buoyancy, grit, and reduces boredom, aiding engagement and persistence in language learning (Cheng, 2023; Fathi et al., 2023; Mohammad Hosseini et al., 2023). It lowers anxiety and boredom, enhances creativity, and makes learning more enjoyable. By fostering non-judgmental awareness, mindfulness keeps students engaged during challenging tasks, improving language acquisition. It also mediates academic demands, helping students in high-stress settings avoid burnout and sustain engagement (Wu and Zhao, 2023).

Overall, mindfulness enhances cognitive functions, reduces anxiety, and increases engagement, all critical for language learning success (Kabat-Zinn, 2015; Zeilhofer and Sasao, 2022). As research explores its mechanisms, mindfulness is poised to become a key educational strategy for improving performance and well-being.

### 2.5 Theoretical framework and hypothesized model

This study examines the relationships among mindfulness, grit, self-regulation, and L2 boredom in Chinese undergraduate English majors, using a mixed-methods approach to understand how these constructs shape L2 classroom experiences. The selection of mindfulness, grit, and self-regulation as predictors is firmly grounded in contemporary theories emphasizing learner agency and the active management of challenging academic experiences, a perspective particularly salient in L2 learning where boredom frequently arises.

Our theoretical foundation draws primarily from Pekrun’s (2006) Control-Value Theory of Achievement Emotions, which suggests boredom arises from a perceived lack of control or value in tasks. We hypothesize that mindfulness, grit, and self-regulation act as complementary internal resources learners use to directly counter these boredom triggers. For example, mindfulness (Kabat-Zinn, 2015; Shapiro et al., 2006) enhances attentional control and adaptive emotional appraisals, helping learners re-engage with uninteresting tasks. Grit (Duckworth et al., 2007) provides the passion and perseverance needed to pursue long-term goals despite immediate monotony, addressing the value component of boredom. Self-regulation (Zimmerman, 2000; Boekaerts and Cascallar, 2006) offers practical strategies to exert control, manage difficulties, and actively create or perceive value in learning.

**FIGURE 1:**
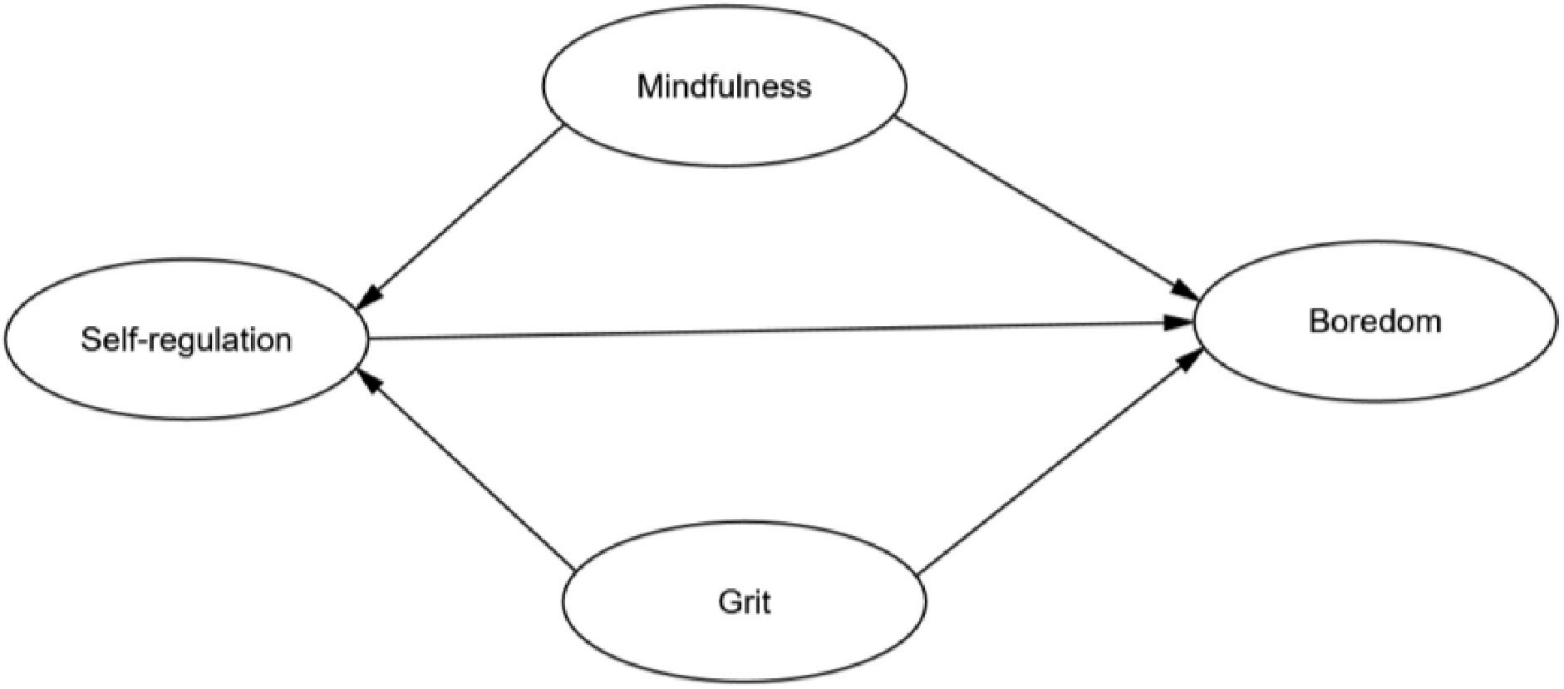
The hypothesized model.

Our hypothesized model (see Figure 1) proposes that mindfulness and grit will negatively predict L2 boredom, with self-regulation mediating these relationships. Examining these constructs collectively allows for a comprehensive understanding of their relative importance and potential interplay in mitigating L2 boredom. Self-regulation is conceptualized as a key mechanism through which the more dispositional qualities of mindfulness and grit are translated into concrete, boredom-reducing actions.

This mixed-methods study is guided by the following research questions:

1. What are the relative direct effects of mindfulness, grit, and self-regulation on L2 boredom?
2. Does self-regulation mediate the relationships between mindfulness and grit with L2 boredom when considered together?
3. How do students experience the interplay of mindfulness, grit, self-regulation, and L2 boredom in English language learning?

Based on the theoretical framework and prior research, this study proposes the following hypotheses for the quantitative phase:

- H1: Mindfulness will be negatively associated with L2 boredom.
- H2: Grit will be negatively associated with L2 boredom.
- H3: Self-regulation will be negatively associated with L2 boredom.
- H4: Self-regulation will mediate the relationship between mindfulness and L2 boredom.
- H5: Self-regulation will mediate the relationship between grit and L2 boredom.

The quantitative phase of this study tests this proposed model, drawing on principles from educational psychology and language learning research. It investigates the direct effects of mindfulness and grit on L2 boredom, expecting mindfulness to reduce boredom by enhancing cognitive and emotional regulation (Kabat-Zinn, 2003; Zeilhofer and Sasao, 2022), and grit to lower boredom through fostering persistence and sustained motivation (Duckworth et al., 2007; Teimouri et al., 2022). Crucially, this phase also explores whether self-regulation mediates these relationships, testing the hypothesis that mindful and gritty students use self-regulatory strategies—like goal setting and self-monitoring—to mitigate boredom (Oxford, 2016; Zimmerman, 2000). Furthermore, we assess if these patterns remain consistent across different demographic groups, including gender and years of English learning experience.

The qualitative phase, conducted through focus group discussions, offers deeper insight into students’ lived perceptions of the interplay among mindfulness, grit, self-regulation, and L2 boredom in their English language learning. This phase examines how students practically apply these constructs to manage boredom, how grit supports persistence, and how the classroom environment and teaching methods influence engagement. By blending quantitative and qualitative methods, this study robustly addresses existing literature gaps, exploring both statistical relationships and contextual influences on classroom boredom. This comprehensive approach ensures our findings advance theoretical knowledge of psychological factors in L2 boredom and provide practically informed guidance for educators.

## 3 Materials and methods

This study employed a mixed methods design, integrating quantitative and qualitative approaches to provide a comprehensive understanding of the relationships among mindfulness, grit, self-regulation, and L2 boredom (Creswell and Plano Clark, 2018). The quantitative phase involved administering structured questionnaires to a large sample of undergraduate English majors, while the qualitative phase included focus groups to gain deeper insights into students’ experiences. This design allows for the triangulation of data, enhancing the validity and richness of the findings (Tashakkori and Teddlie, 2010).

### 3.1 Participants

The study involved 516 undergraduate English majors from various universities in Mainland China. Participants were chosen through a multistage random sampling process to ensure diversity and representativeness. Initially, 20 universities offering English major programs were randomly selected from a comprehensive list using a random number generator. These universities were spread across six provinces to reflect a broad geographical representation.

Within each selected university, a stratified random sampling method was employed based on academic year (freshman to senior) to ensure that students at different levels of their academic journey were included. Random sampling within each group was conducted using software like SPSS, resulting in a diverse range of students with varying levels of exposure to English language learning. The participants ranged from 18 to 24 years old, with an average age of 20.7 years (SD = 1.4). The gender distribution was 57% female (294 participants) and 43% male (222 participants), reflecting the typical gender composition in English major programs in China. All participants had studied English as a foreign language for about 10 years (SD = 2.1), beginning in middle school. Their English proficiency was generally at an intermediate level, verified through standardized English test scores (e.g., CET-4), indicating the sample was relatively comparable in terms of baseline language skills for investigating the target psychological variables.

In addition to the quantitative sample, the qualitative phase involved focus groups with a subset of participants to gain deeper insights into their experiences. Forty students were selected through purposive sampling from the main study sample to ensure diverse representation based on gender, academic year, and English proficiency. These participants provided richer, context-specific data to complement the quantitative findings.

Data collection for the quantitative phase occurred in a classroom setting, with participants completing questionnaires during class hours under supervision. Prior to participation, all students were informed about the study’s purpose, voluntary nature, and confidentiality assurances. Informed consent was obtained in accordance with ethical guidelines. The research protocol was reviewed and approved by the Ethics Committee of Yulin Normal University, ensuring adherence to ethical standards for research involving human participants.

### 3.2 Instruments

To assess the key constructs of mindfulness, grit, self-regulation, and L2 boredom, a series of well-established psychometric instruments were employed. Each was selected based on demonstrated reliability and validity in similar research contexts, particularly within Chinese educational settings. All instruments were administered in their original English versions, as the participants, being English majors, were proficient enough to comprehend the items without translation.

Mindfulness was measured using the 15-item Five Facet Mindfulness Questionnaire (FFMQ-15; Baer et al., 2012). This scale captures the multifaceted nature of mindfulness (including observing, describing, acting with awareness, non-judging, and non-reactivity) using a 5-point Likert scale (1 = *never or very rarely true* to 5 = *very often or always true*), where higher scores indicate greater mindfulness. In this study, the FFMQ-15 demonstrated acceptable internal consistency (Cronbach’s α = 0.82) and Confirmatory Factor Analysis (CFA) indicated good model fit (χ^2^/df = 2.31, CFI = 0.95, TLI = 0.93, RMSEA = 0.052 [90% *CI* = 0.045, 0.060], SRMR = 0.034).

Self-regulation was assessed with the 31-item Short Self-Regulation Questionnaire (SSRQ; Carey et al., 2004), covering dimensions such as goal setting, planning, self-monitoring, and perseverance. Participants responded on a 5-point Likert scale (1 = *strongly disagree* to 5 = *strongly agree*), with higher scores reflecting stronger self-regulatory abilities. The SSRQ exhibited excellent internal consistency (Cronbach’s α = 0.92) and good CFA model fit in this sample (χ^2^/df = 2.45, CFI = 0.94, TLI = 0.92, RMSEA = 0.056 [90% *CI* = 0.048, 0.064], SRMR = 0.036).

L2 boredom was measured using the 8-item Foreign Language Classroom Boredom Subscale (FLCBS), part of the Foreign Language Learning Boredom Scale (FLLBS; Li et al., 2023). This subscale assesses boredom experienced during English classes (e.g., “I am only physically in the classroom, while my mind is wandering outside the English class”) on a 5-point Likert scale (1 = *strongly disagree* to 5 = *strongly agree*). The FLCBS showed high internal consistency (Cronbach’s α = 0.93) and satisfactory CFA model fit (χ^2^/df = 1.98, CFI = 0.96, TLI = 0.94, RMSEA = 0.048 [90% *CI* = 0.041, 0.056], SRMR = 0.032).

L2 grit was measured using a 10-item scale adapted from Lee (2020) that captures perseverance of effort and consistency of interest regarding long-term English learning goals. Responses were given on a 5-point Likert scale (1 = *“not like me at all”* to 5 = *“very much like me”*). The scale yielded high internal consistency (Cronbach’s α = 0.89). Additionally, CFA results supported its construct validity with good model fit (χ^2^/df = 2.19, CFI = 0.95, TLI = 0.93, RMSEA = 0.051 [90% *CI* = 0.044, 0.059], SRMR = 0.035) and significant factor loadings ranging from 0.65 to 0.85.

For the qualitative phase, focus group discussions explored participants’ experiences with these constructs. Forty students were purposefully selected from the main sample to ensure diversity based on gender, academic year, and English proficiency, aiming to capture a wide range of perspectives. Four focus group sessions, each with 10 participants, were conducted in a quiet campus setting and lasted approximately 60 min to foster comfortable and open discussion.

A semi-structured interview guide directed the focus groups, featuring questions designed to elicit participants’ strategies for managing boredom and maintaining engagement in English classes (e.g., “Can you describe how you cope with boredom in your English classes?”). This approach complemented the quantitative findings by exploring the constructs in greater depth. With participants’ consent, discussions were audio-recorded and later transcribed verbatim for thematic analysis.

### 3.3 Procedure

Data collection for the quantitative phase was conducted over 6 weeks during the spring semester, avoiding periods with major exams to minimize external stressors that could affect responses. Sessions were scheduled during regular class hours to optimize student alertness. The environment was set up to ensure privacy and minimize disruptions, with participants seated apart and instructed to focus solely on the questionnaires.

All questionnaires were completed in a single 40-min session. To maintain participant concentration and data quality, instructions were provided before each questionnaire. A hybrid data collection approach was used, offering both online and paper formats. Preliminary analysis indicated no significant differences in responses between formats, ensuring consistency in data collection. Focus group sessions were held separately from the quantitative data collection. A trained moderator facilitated each session using the semi-structured interview guide. Audio recordings were securely stored, and confidentiality was maintained throughout the process.

### 3.4 Data analysis

This study used a mixed methods approach, combining quantitative and qualitative techniques to examine relationships among mindfulness, grit, self-regulation, and L2 boredom. Quantitative analysis started with data screening in SPSS version 26, generating descriptive statistics. Missing values (<5% per variable) were addressed using the Expectation-Maximization algorithm (Tabachnick and Fidell, 2019). Outliers, detected via Mahalanobis distance, were removed if extreme and unjustifiable to maintain data integrity. Normality was verified with *skewness* and *kurtosis* statistics, all within acceptable ranges (| <*skewness*> | <2, | <*kurtosis*> | <7), confirming suitability for SEM (West, 1995).

Descriptive statistics (*M*, *SD*, Pearson correlations) outlined sample traits and variable relationships. Cronbach’s alpha assessed internal consistency, with all scales exceeding 0.70, indicating reliability (Nunnally and Bernstein, 1994). SEM in AMOS version 24 validated the measurement model via Confirmatory Factor Analysis (CFA), testing observed variables against latent constructs. Fit indices—*χ^2^*, CFI, TLI, RMSEA, and SRMR—showed good fit (CFI and TLI > 0.90, RMSEA and SRMR < 0.08) (Hu and Bentler, 1999). Factor loadings, all significant and above 0.50, supported convergent validity.

The structural model tested hypothesized relationships, fitting well and revealing mindfulness and self-regulation as strong negative predictors of L2 boredom. Grit also negatively related to boredom, though less strongly, suggesting mindfulness and self-regulation play larger roles in reducing it. Mediation analysis, using the bootstrap method with 5,000 resamples (Preacher and Hayes, 2008), confirmed that self-regulation partially mediates the relationships between both mindfulness and L2 boredom, and grit and L2 boredom, underscoring its role as a key mechanism. Multi-group analysis across gender and years of English learning found no significant differences, indicating consistent relationships across subgroups (Cheung and Rensvold, 2002).

Qualitative analysis involved thematic analysis of focus group data, guided by Braun and Clarke (2006), to explore students’ experiences with these constructs. Audio-recorded discussions were transcribed, checked for accuracy, coded for recurring ideas, and categorized into themes reflecting participants’ views, deepening insight into real learning contexts.

Integrating quantitative and qualitative findings enriched the analysis. Qualitative data provided context for quantitative results, showing how students use mindfulness and self-regulation to manage boredom in L2 learning. This mixed methods design offered a thorough examination of the relationships, with triangulation enhancing validity and depth.

## 4 Results

### 4.1 Quantitative results

#### 4.1.1 Descriptive statistics and correlational analyses

Table 1 presents descriptive statistics and correlations among the key variables: mindfulness, grit, self-regulation, and L2 boredom. Participants exhibited moderate to high levels of mindfulness (*M* = 3.72, SD = 0.58) and grit (*M* = 3.85, SD = 0.61), with self-regulation being the highest (*M* = 3.94, SD = 0.57). L2 boredom was moderate (*M* = 2.85, SD = 0.72).

**TABLE 1:** Descriptive statistics and intercorrelations among study variables.

Variable	Mean	SD	1	2	3	4
1. Mindfulness	3.72	0.58	–			
2. Grit	3.85	0.61	0.29***	–		
3. Self-regulation	3.94	0.57	0.46***	0.38***	–	
4. L2 boredom	2.85	0.72	−0.42***	−0.34***	−0.51***	–

All correlations are significant at the ***p < 0.001 level.

As hypothesized, mindfulness and self-regulation were negatively correlated with L2 boredom (*r* = −0.42 and *r* = −0.51, respectively, *p* < 0.001). Grit also showed a significant, though weaker, negative correlation with boredom (*r* = −0.34, *p* < 0.001). Additionally, positive correlations were found between mindfulness and grit (*r* = 0.29, *p* < 0.001), mindfulness and self-regulation (*r* = 0.46, *p* < 0.001), and grit and self-regulation (*r* = 0.38, *p* < 0.001). These results suggest that students who are more mindful and gritty tend to also be more self-regulated, and that all three traits are associated with lower levels of L2 boredom.

#### 4.1.2 Measurement model

A Confirmatory Factor Analysis (CFA) was conducted using AMOS version 24 to assess the validity and reliability of mindfulness, grit, self-regulation, and L2 boredom. The measurement model demonstrated a very good fit with the data. Key fit indices included a Chi-square (χ^2^) of 211.34 with 123 degrees of freedom, and a relative Chi-square (χ^2^/df) of 1.72. While the Chi-square was significant (*p* < 0.001) due to the large sample size, other indices provided stronger evidence of a good fit: CFI was 0.96, TLI was 0.94, RMSEA was 0.043 (90% CI = 0.036 to 0.051), and SRMR was 0.032, all within acceptable ranges (Hu and Bentler, 1999; Browne and Cudeck, 1993).

The factor loadings for all items on their respective constructs were significant at *p* < 0.001, indicating strong relationships between the indicators and their underlying factors. Specifically, mindfulness loadings ranged from 0.70 to 0.82, grit loadings ranged from 0.65 to 0.85, self-regulation loadings ranged from 0.72 to 0.88, and L2 boredom loadings ranged from 0.62 to 0.80.

Convergent validity, as shown in Table 2, was established with AVE values exceeding 0.50 for all constructs (Fornell and Larcker, 1981). Discriminant validity was confirmed by comparing the square root of the AVE for each construct with its correlations with other constructs. The square roots of the AVEs were greater than any inter-construct correlations, ranging from 0.29 to 0.51, indicating clear distinctiveness between the constructs. Reliability was further supported by Cronbach’s alpha and Composite Reliability (CR) values, all exceeding the 0.70 threshold, demonstrating strong internal consistency (Nunnally and Bernstein, 1994). The CFA results, therefore, validated the measurement model, indicating that mindfulness, grit, self-regulation, and L2 boredom are accurately represented by their respective indicators. Both convergent and discriminant validity were firmly established, ensuring the constructs’ measurement integrity and providing a solid foundation for subsequent analysis.

**TABLE 2:** Convergent and discriminant validity.

Construct	AVE	Square root of AVE	Cronbach’s alpha	Composite reliability
Mindfulness	0.65	0.81	0.82	0.84
Grit	0.68	0.82	0.89	0.91
Self-regulation	0.72	0.85	0.92	0.93
L2 boredom	0.61	0.78	0.86	0.88

The square root of the AVE is greater than the highest inter-construct correlations, confirming discriminant validity.

#### 4.1.3 Structural equation modeling (SEM)

**FIGURE 2:**
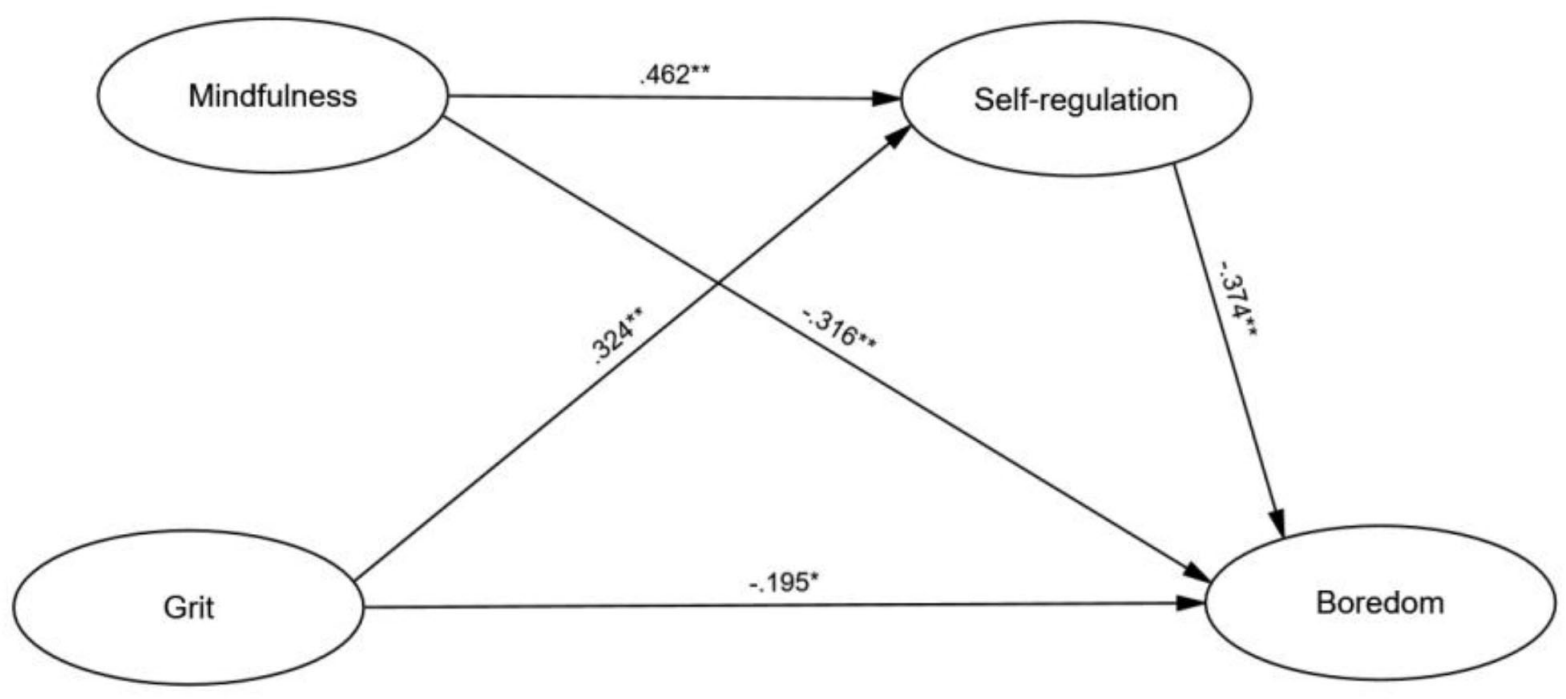
Structural model for the relationships among mindfulness, grit, self-regulation, and L2 boredom. Standardized path coefficients (β) are displayed. *p < 0.01, **p < 0.001.

Structural equation modeling was employed to test the hypothesized model, examining the direct effects of mindfulness, grit, and self-regulation on L2 boredom. This approach allows for the assessment of complex relationships while accounting for measurement error, offering a robust analysis. The model demonstrated an excellent fit to the data (χ^2^/df = 1.80, CFI = 0.95, TLI = 0.93, RMSEA = 0.045, SRMR = 0.035), supporting its adequacy in representing the relationships among the variables (Hu and Bentler, 1999; Browne and Cudeck, 1993; Kline, 2015). The SEM results revealed significant direct effects of mindfulness, grit, and self-regulation on L2 boredom. These path coefficients are illustrated in Figure 2 and shown in Table 3.

**TABLE 3:** Standardized path coefficients for direct effects in the structural model.

Path	β (standardized coefficient)	SE	*p*-value
Mindfulness → L2 boredom	−0.316	0.04	<0.001
Mindfulness → self-regulation	0.462	0.05	<0.001
Grit → self-regulation	0.324	0.05	<0.001
Grit → L2 boredom	−0.195	0.02	<0.01
Self-regulation → L2 boredom	−0.374	0.04	<0.001

Mindfulness had a significant negative effect on L2 boredom (β = −0.316, *p* < 0.001), indicating that higher mindfulness is linked to less boredom in language learning. Self-regulation showed a stronger negative effect on L2 boredom (β = −0.374, *p* < 0.001), emphasizing its role in managing engagement. Grit also negatively impacted L2 boredom (β = −0.195, *p* < 0.01), although its effect was smaller than mindfulness and self-regulation.

#### 4.1.4 Mediation analysis

To further elucidate the relationships between these variables, a mediation analysis was conducted to test whether self-regulation mediates the relationship between mindfulness and L2 boredom. The analysis utilized the bootstrap method with 5,000 resamples, which is a robust approach for assessing indirect effects (Preacher and Hayes, 2008). The results of the mediation analysis are presented in Table 4.

**TABLE 4:** Standardized path coefficients for indirect effects in the mediation model.

Indirect path	β (standardized coefficient)	SE	95% CI	*p*-value
Mindfulness → self-regulation → L2 boredom	−0.173	0.04	[−0.238, −0.111]	<0.001
Grit → self-regulation → L2 boredom	−0.121	0.03	[−0.178, −0.068]	<0.001

The analysis found significant indirect effects of mindfulness on L2 boredom through self-regulation (β = −0.173, *p* < 0.001) and grit on L2 boredom through self-regulation (β = −0.121, *p* < 0.001). These findings indicate that self-regulation partially mediates the impact of both mindfulness and grit on L2 boredom.

#### 4.1.5 Multi-group analysis

A multi-group analysis was performed to examine if the relationships between mindfulness, grit, self-regulation, and L2 boredom varied by gender or years of English learning experience. The sample was divided by gender (male: *n* = 222, female: *n* = 294) and years of English learning (low: 6–8 years, *n* = 134; medium: 9–11 years, *n* = 262; high: 12–14 years, *n* = 120).

Baseline models for each group were first estimated freely. Then, structural paths were constrained to be equal across groups to test for invariance. Fit indices for both unconstrained and constrained models are shown in Table 5.

**TABLE 5:** Fit indices for multi-group analysis across gender and years of English learning experience.

Model	χ^2^	df	CFI	TLI	RMSEA	SRMR	Δχ^2^ (Δdf)	*p*-value
Unconstrained (gender)	410.28	246	0.94	0.92	0.044	0.036	–	–
Constrained (gender)	419.56	253	0.94	0.92	0.043	0.038	9.28 (7)	0.23
Unconstrained (years of learning)	622.14	372	0.93	0.91	0.046	0.039	–	–
Constrained (years of learning)	634.47	383	0.93	0.91	0.045	0.040	12.33 (11)	0.34

The unconstrained models showed good fit for both gender and years of English learning experience, with CFI > 0.90, TLI > 0.90, RMSEA < 0.05, and SRMR < 0.08. The Chi-square difference tests (Δχ^2^) for both gender (Δχ^2^ = 9.28, Δdf = 7, *p* = 0.23) and years of learning (Δχ^2^ = 12.33, Δdf = 11, *p* = 0.34) were not significant, indicating that the structural paths are invariant across these groups.

These findings suggest that the relationships among mindfulness, grit, self-regulation, and L2 boredom are consistent regardless of gender and English learning experience. The direct effects of mindfulness, grit, and self-regulation on L2 boredom, along with the mediation by self-regulation, apply similarly across different demographic subgroups.

Overall, the quantitative data offers clear evidence that mindfulness, grit, and self-regulation are significant predictors of L2 boredom in Chinese EFL students. Among these, mindfulness and self-regulation emerged as the most influential factors, with mindfulness also exerting an indirect effect on L2 boredom via self-regulation. Although grit was a significant predictor, its impact was less pronounced. These results remained consistent across gender and different durations of English learning experience, indicating the wide applicability of these psychological constructs in mitigating boredom in language learning.

### 4.2 Qualitative results

The focus group discussions provided rich, contextualized insights into students’ lived experiences of L2 boredom, mindfulness, grit, and self-regulation in English language learning. These qualitative data directly address Research Question 3 – How do students experience the interplay of these constructs in English language learning? – and offer a deeper understanding that complements the quantitative findings by highlighting the emotional, cognitive, and behavioral dimensions of their learning process. The thematic analysis revealed three core areas: the multifaceted nature of L2 boredom, the perceived role of individual psychological resources (mindfulness, grit, self-regulation) in managing it, and the influence of the teacher and classroom environment.

#### 4.2.1 The multifaceted nature of L2 boredom

In response to Research Question 3, students consistently described L2 boredom not as a singular feeling, but as a complex, layered experience encompassing emotional, cognitive, and behavioral dimensions that profoundly led to disengagement and dissatisfaction. This rich description forms the foundational context for understanding how other psychological resources are brought into play to manage this experience.

Emotionally, boredom manifested as a sense of emptiness or lack of purpose. Participants articulated feeling an overwhelming apathy toward the learning material. As one student shared, “It’s like being stuck in a waiting room, endlessly watching the clock tick, with no sense of purpose or excitement.” This emotional detachment created an unmotivated state, suggesting boredom was more than a mild annoyance; it was a deep internal disconnect.

Cognitively, students frequently reported a “mental fog” that severely impaired their ability to concentrate and process information. Thoughts would drift aimlessly, pulling their attention away from the lesson. A common sentiment was, “Sometimes, I realize that I’ve missed entire sections of the lecture because my mind was elsewhere.” This intellectual disengagement underscored a significant barrier to effective learning, directly impacting comprehension and retention.

Behaviorally, boredom triggered overt actions aimed at self-stimulation or distraction. Students described fidgeting, doodling, or habitually checking their phones. A freshman elaborated, “I start tapping my foot or playing with my pen. It’s like my body is trying to find something to do because my mind is so bored.” Another admitted, “I’ll glance at my phone every few minutes, even if there’s nothing new. It’s just a way to break the monotony.” These physical manifestations highlighted a restless attempt to cope with an unengaging classroom environment. This detailed qualitative picture of boredom, as a state deeply affecting emotional, cognitive, and behavioral engagement, aligns with the moderate level of L2 boredom observed in our quantitative data (*M* = 2.85, SD = 0.72), affirming its real and impactful presence for learners.

#### 4.2.2 Individual psychological resources in managing L2 boredom

Students’ narratives provided rich accounts of how they perceived and leveraged mindfulness, grit, and self-regulation as internal resources to navigate and reduce L2 boredom. The discussions illuminated the interplay of these constructs as students actively sought to maintain engagement.

#### 4.2.3 Mindfulness: an anchor for present focus

Participants consistently viewed mindfulness as an essential tool for maintaining focus and emotional balance in class, serving as a “stabilizing force” amidst internal and external distractions. A junior student aptly described it as, “Mindfulness is like a quiet space in the middle of a storm. It helps me find my center and focus, even when everything around me feels chaotic.” Students explained that simple techniques, such as mindful breathing or focusing on bodily sensations, allowed them to consciously re-engage with the present moment. A senior noted, “It’s like I’m waking up from a dream. Mindfulness helps me to snap out of the boredom and realize that I’m actually in class, and there’s something to learn here.” Significantly, students emphasized that mindfulness didn’t eliminate distractions but rather helped them observe these distractions without frustration, enabling a gentle return to the task. This non-judgmental acceptance, as a sophomore put it, “is not about forcing myself to be interested, but about accepting that my mind will wander sometimes. Mindfulness helps me notice that and bring my focus back without getting frustrated.” These reflections underscore how mindfulness fosters self-compassion and acceptance, transforming disengagement into an opportunity for re-focus, which directly supports the quantitative finding of a significant negative effect of mindfulness on L2 boredom (β = −0.316, *p* < 0.001).

#### 4.2.4 Grit: fueling long-term perseverance

Grit emerged as a powerful motivator that enabled students to persist through learning challenges and monotony. They characterized grit as an “internal flame” fueling their determination even when progress was slow or immediate rewards were absent. One junior student likened learning English to climbing a mountain, stating: “There are days when it feels like every step is an uphill battle, and the summit is nowhere in sight. But grit keeps me moving, one step at a time, even when I’m tired and the view is obscured.” This highlights a deep commitment to long-term goals that transcends momentary boredom. Students emphasized consistent effort over innate talent, with a senior male observing, “It’s not about being a natural language learner. It’s about showing up, day after day, even when it’s the last thing you want to do. That’s where grit comes in.” Furthermore, participants spoke about reframing boredom or challenges as tests of their resolve, transforming potential quitting points into opportunities for growth. As a freshman male shared, “When I feel bored or discouraged, I tell myself that this is just part of the journey. If I can push through this, I’ll come out stronger on the other side.” These insights strongly complement the quantitative finding that grit negatively predicts L2 boredom (β = −0.195, *p* < 0.01), illustrating how grit facilitates persistence despite unstimulating tasks.

#### 4.2.5 Self-regulation: a proactive toolkit for engagement

Students consistently perceived self-regulation as a dynamic set of practical strategies they actively employed to maintain focus and motivation, effectively navigating the complexities of language learning. A female senior described it as akin to “having a map and compass in a dense forest,” suggesting its critical role in guided learning. Key strategies included setting clear, achievable goals and breaking down larger tasks into manageable steps. A male junior explained, “I break down big assignments into smaller steps. It’s like eating an elephant one bite at a time. It makes the task seem less intimidating and helps me avoid procrastination.” To sustain motivation, students also highlighted the use of self-reward systems: “I treat myself to something I enjoy after completing a difficult task. It’s a way to acknowledge my hard work and make the process more enjoyable,” a sophomore female noted. Other mentioned techniques included creating structured study schedules and utilizing positive self-talk. A male junior stated, “I set specific times for studying and stick to them. It creates a routine that helps me stay disciplined,” while a female freshman emphasized, “When I start to lose focus, I remind myself why I’m learning English. It helps to have that internal dialogue to stay motivated.” These proactive behaviors illustrate how students become active agents in managing their learning and emotions, directly reflecting the strong negative effect of self-regulation on L2 boredom (β = −0.374, *p* < 0.001). Importantly, the discussions showed how students often used self-regulatory actions to apply their mindfulness (e.g., using a self-monitoring strategy to notice mind-wandering) and enact their grit (e.g., planning and persisting on difficult tasks), providing rich qualitative support for self-regulation’s mediating role observed quantitatively.

#### 4.2.6 The influence of external factors: teacher and classroom environment

Beyond individual psychological resources, the qualitative data highlighted the crucial role of teachers and the classroom environment in shaping students’ boredom and engagement levels, emerging as a significant contextual factor in their learning experiences.

Students strongly emphasized that teachers who utilized dynamic and interactive teaching methods served as catalysts for interest. A female sophomore articulated this vividly: “A good teacher is like a master chef, blending different flavors to create a delicious and satisfying meal. They don’t just serve the same dish every day; they keep us guessing and wanting more.” Participants particularly valued activities that encouraged active participation, such as group discussions and hands-on exercises, believing they fostered a deeper connection to the material: “I learn best by doing, not just by listening. When we have interactive activities, I feel more connected to the material and less likely to get bored,” shared a female senior.

Furthermore, a supportive classroom atmosphere was deemed essential. A male junior noted, “When the teacher creates a safe space where we can express ourselves without fear of judgment, it makes learning more enjoyable and less boring.” Conversely, monotonous lectures and repetitive tasks consistently led to disengagement. A male freshman expressed, “It’s like watching paint dry. When the teacher just drones on and on, and we have no chance to contribute, it’s impossible not to get bored.” Another student lamented the lack of active involvement: “Sometimes, it feels like we’re just there to listen and take notes, but we’re not really involved in the learning process. That makes it really hard to stay focused and engaged” (Female, Junior).

These insights reveal that while individual psychological resources are vital, pedagogical practices and the learning environment significantly impact whether boredom is mitigated or exacerbated, suggesting that teacher behaviors can facilitate or hinder the application of students’ mindfulness, grit, and self-regulation.

## 5 Discussion

This mixed-methods study explored the complex relationships among mindfulness, grit, self-regulation, and L2 boredom among Chinese undergraduate English majors. By integrating quantitative data with qualitative insights from focus group discussions, the study provides a comprehensive understanding of how these psychological constructs interact to influence students’ experiences in language learning.

Quantitative findings revealed mindfulness significantly and negatively related to L2 boredom. This aligns with existing literature on mindfulness’s role in emotional regulation, attention control, and academic engagement (Kabat-Zinn, 2015; Zeilhofer and Sasao, 2022; Morgan and Katz, 2021). Mindfulness practices help students maintain focus through non-judgmental present-moment awareness (Shapiro et al., 2006; Kabat-Zinn, 2003), reducing disengagement. This is especially vital in L2 learning for sustained attention and engagement (Ghanizadeh et al., 2019; Zeilhofer, 2023), as mindfulness enhances cognitive function and reduces mind-wandering, improving language processing (Fathi et al., 2023).

The qualitative data enriched this understanding by illustrating how students perceive and utilize mindfulness to manage boredom. Participants described mindfulness as an “anchor” that helps them maintain focus amid distractions. One junior student remarked, “Mindfulness is like a quiet space in the middle of a storm. It helps me find my center and focus, even when everything around me feels chaotic.” This deep resonance with Kabat-Zinn’s (2015) concept of present-moment awareness directly counters boredom’s disengagement as theorized by Control-Value Theory (Pekrun, 2006). By cultivating non-judgmental acceptance of tasks and emotions, learners adapt their appraisals of task value and difficulty, regaining control and reducing boredom’s emotional impact. This mindful acceptance fosters emotional regulation, decreasing negative states like anxiety and stress (Khoury et al., 2013; Morgan and Katz, 2021; Ghanizadeh et al., 2019) that often exacerbate boredom (Pekrun et al., 2010). Similarly, grit consistently linked negatively with L2 boredom, reinforcing its vital role in sustaining engagement (Duckworth et al., 2007; Teimouri et al., 2022). Gritty students are less prone to boredom due to their persistent, goal-oriented nature (Datu et al., 2017; Wei et al., 2020). From Control-Value Theory, grit directly addresses boredom’s “value” component: learners maintain commitment to long-term goals despite immediate lack of interest, finding enduring value beyond transient disengagement. This trait builds resilience, helping students persist through setbacks and maintain long-term interest (Eskreis-Winkler et al., 2014). The qualitative data further illuminated this, with students describing grit as an “internal flame” driving their determination. One student compared English learning to mountain climbing: “There are days when it feels like every step is an uphill battle. But grit keeps me moving, one step at a time.” This shows how gritty students stay focused on their ultimate goal, seeing obstacles as temporary. Their ability to maintain effort even through uninteresting tasks directly counteracts boredom. Moreover, L2 grit mitigates foreign language anxiety, serving as a crucial resource for achievement across diverse learner profiles (Liu et al., 2025a) and mediating the impact of perceived teacher support on anxiety (Liu et al., 2025b). This expanded understanding emphasizes grit’s comprehensive role in navigating L2 learning’s emotional and motivational challenges.

Furthermore, research indicates that grit is associated with higher levels of self-regulation and self-discipline, which help students engage in deliberate practice and stay committed to their learning goals (Duckworth and Gross, 2014; Alamer, 2022). In the context of L2 learning, gritty students are more likely to employ effective learning strategies, seek out challenging materials, and persist in the face of difficulties, thereby reducing experiences of boredom (Teimouri et al., 2022; Lee, 2022). This persistence aligns with Dörnyei et al. (2015), who emphasize the importance of motivation and self-regulation in sustaining language learning efforts over time.

Self-regulation emerged as a significant mediator between both mindfulness and grit, and L2 boredom. Quantitatively, self-regulation partially mediated the relationship between mindfulness and L2 boredom (indirect effect: β = −0.173, *p* < 0.001), accounting for approximately 35% of the total effect. This substantial indirect pathway highlights that mindfulness’s positive influence on reducing boredom is significantly channeled through enhanced self-regulatory behaviors. This aligns with Social Cognitive Theory (Bandura, 1986) and Self-Regulated Learning models (Zimmerman, 2000), particularly by demonstrating how mindfulness improves executive functions such as attention control, emotional regulation, and metacognitive awareness (Shapiro et al., 2006; Schunk and Zimmerman, 2023). These cognitive and metacognitive processes are fundamental to the forethought, performance, and self-reflection phases of Zimmerman’s cyclical model, enabling learners to actively manage their engagement and directly exert control over their learning environment, thus reducing boredom. By fostering these self-regulatory skills, mindfulness helps students stay focused on their learning objectives, reducing the likelihood of disengagement and boredom (Fathi et al., 2023; Shapiro et al., 2006).

Similarly, self-regulation also mediated the relationship between grit and L2 boredom (indirect effect: β = −0.121, *p* < 0.001), explaining approximately 38% of the total effect. While this indirect effect is statistically significant, the direct effect of grit (β = −0.195) remained notable, suggesting grit influences boredom both directly and indirectly via self-regulation. This finding further reinforces the interconnectedness proposed by Social Cognitive Theory, where grit (as a motivational trait) translates into tangible self-regulatory actions and persistence (Duckworth and Gross, 2014). Gritty individuals, through their commitment to long-term goals, are more likely to engage in the planning, monitoring, and strategic adjustments central to self-regulation (Credé et al., 2017; Eskreis-Winkler et al., 2014). This active engagement provides learners with a sense of control over their learning process, directly combating the perceived lack of control that precipitates boredom according to Control-Value Theory. In the context of language learning, this means that students with higher grit are better equipped to employ effective learning strategies, manage challenges, and stay engaged, thereby reducing experiences of boredom (Alamer, 2021; Lee, 2022; Teimouri et al., 2022).

A crucial consideration, however, is the conceptual overlap between grit and self-regulation (Duckworth and Gross, 2014). In our model, self-regulation exhibited a stronger direct negative relationship with L2 boredom and significantly mediated the grit-boredom link. This raises questions about grit’s unique contribution, particularly its “perseverance of effort” dimension, beyond effective self-regulatory strategies (Credé, 2018). While grit’s direct effect remained significant—suggesting it may capture aspects like “consistency of interest” not fully measured by self-regulation—our findings align with views that a substantial part of grit’s influence on academic outcomes operates through, or is closely tied to, core self-regulatory functions.

Qualitatively, students described self-regulation as a practical toolkit for maintaining focus and motivation. They employed strategies like setting clear goals and breaking down large tasks, as one student explained, “I break down big assignments into smaller steps. It’s like eating an elephant one bite at a time. It makes the task seem less intimidating and helps me avoid procrastination.” Students also used self-reward systems to sustain motivation, treating themselves “to something I enjoy after completing a difficult task.” This proactive approach, reflecting Boekaerts and Cascallar’s (2006) emphasis on motivational strategies, allows students to counteract boredom by actively creating a more engaging and meaningful learning experience. Effective self-regulation enhances learners’ autonomy and competence, key factors in maintaining intrinsic motivation and reducing boredom (Greene, 2017; Schunk and Zimmerman, 2023; Zimmerman, 2002).

The significant impact of teaching methods and the classroom environment on student engagement was also highlighted in the qualitative findings. Students emphasized the teacher’s role as a catalyst for engagement, noting that dynamic and interactive teaching methods enhance interest and reduce boredom. One student remarked, “A good teacher is like a master chef, blending different flavors to create a delicious and satisfying meal. They don’t just serve the same dish every day; they keep us guessing and wanting more.” This underscores the importance of creativity and adaptability in teaching, aligning with Dörnyei’s (2009) emphasis on motivational teaching practices and Oxford’s (2016) advocacy for dynamic instructional methods.

From a theoretical standpoint, effective teaching practices can directly influence the “control” and “value” appraisals central to Control-Value Theory (Pekrun, 2006). Teachers who provide varied instructional strategies, clear scaffolding, and foster a supportive atmosphere can enhance students’ perceived control over learning tasks and increase the value they ascribe to the material. Research supports the notion that varied instructional strategies, such as collaborative learning, problem-based activities, and the use of multimedia resources, can significantly enhance student engagement and reduce boredom (Oxford, 2016; Sharp et al., 2017). By incorporating diverse teaching methods, educators can cater to different learning styles and keep the content fresh and stimulating (Zhang et al., 2022). Conversely, monotonous lectures and lack of interactive opportunities were associated with increased boredom, reflecting Tze et al.’s (2016) findings that lack of stimulation contributes to disengagement. Integrating these findings, the study highlights that L2 classroom boredom is a multifaceted experience, affecting students emotionally, cognitively, and behaviorally. Emotionally, students described feelings of emptiness and disengagement, aligning with views that boredom involves a lack of psychological involvement, low arousal, and dissatisfaction, negatively impacting motivation (Sharp et al., 2017; Pekrun et al., 2010). Cognitively, students reported a “mental fog” hindering concentration, supporting findings that boredom impairs cognitive processing and language acquisition by reducing attention and interfering with memory (Li, 2022; Li et al., 2023; Pekrun, 2006). These cognitive and emotional facets strongly align with Control-Value Theory (Pekrun, 2006), which posits boredom arises from appraisals of low control and value. Behaviorally, actions like fidgeting and phone-checking illustrated how boredom leads to self-regulatory failures and attempts to seek stimulation, disrupting the learning environment (Eastwood et al., 2012; Boekaerts and Cascallar, 2006).

These multifaceted experiences suggest boredom is not merely transient but a significant barrier requiring comprehensive interventions (Pawlak et al., 2020). Effective strategies should enhance individual psychological resources—such as mindfulness for emotional regulation, grit to sustain motivation despite faltering perceived value or control, and self-regulation to manage learning behaviors by enacting control and fostering value. Additionally, optimizing instructional practices and classroom environments is crucial (Pawlak et al., 2022). By adopting this holistic approach, educators can better support students in maintaining engagement and achieving language learning success (Dörnyei, 2009; Oxford, 2016).

## 6 Conclusion

The findings of this study have important theoretical and practical implications for understanding and addressing boredom in L2 learning. Theoretically, the research extends models of language learning motivation by incorporating mindfulness and grit—constructs often overlooked in L2 studies. By demonstrating how mindfulness and grit influence L2 boredom, the study proposes a more comprehensive model that includes cognitive, affective, metacognitive, and self-regulatory processes. Furthermore, identifying self-regulation as a mediator between mindfulness, grit, and L2 boredom supports social cognitive theory (Bandura, 1986) and self-regulated learning models (Zimmerman, 2000), emphasizing the role of self-regulatory behaviors in managing emotional experiences and shaping student engagement.

The qualitative findings provide contextualized insights into how students perceive and apply mindfulness, grit, and self-regulation in their learning. Themes like mindfulness as an anchor, grit as fuel for perseverance, and self-regulation as a toolkit for engagement deepen our understanding of how these constructs function in classroom settings. These insights enrich theoretical frameworks and support the development of holistic, context-sensitive theories of language learning that account for the dynamic nature of student engagement. Collectively, this study significantly enhances theoretical understanding of L2 boredom predictors, particularly by revealing how mindfulness, grit, and self-regulation interact to affect learner engagement.

Practically, the study offers recommendations for educators, curriculum designers, and policymakers. Given the links between mindfulness, grit, self-regulation, and reduced L2 boredom, educators and policymakers should integrate these constructs into language programs through classroom exercises (e.g., mindful breathing), goal-setting workshops, and professional development to enhance student engagement and resilience. These efforts can help students manage emotions, set realistic goals, and persist in challenging tasks. Teachers can further support this by modeling perseverance, using dynamic methods like collaborative learning, and fostering a supportive classroom environment that encourages expression and reduces boredom.

The mixed-methods design reinforces the study’s comprehensive approach, capturing both statistical relationships and student experiences to inform effective interventions. At the policy level, institutions should support these strategies through workshops and teacher training, equipping learners and educators with tools to improve L2 outcomes. By informing interventions to reduce boredom and improve learning environments, the study provides practical insights for educators. The qualitative phase, through focus groups, captures students’ subjective experiences, illustrating how they utilize mindfulness and self-regulation to manage boredom and how grit supports persistence, thereby ensuring interventions align with students’ real needs.

## 7 Limitations and directions for further research

Although this study provides valuable insights into the psychological predictors of L2 boredom, it has limitations. The primary limitation is its cross-sectional design, which, while useful for identifying relationships between variables, does not allow for causal inferences. Future research could employ longitudinal designs to examine how mindfulness, grit, and self-regulation influence L2 boredom over time, offering a more comprehensive understanding of how these constructs develop and interact during the language learning process.

Another limitation is the reliance on self-report measures to assess mindfulness, grit, self-regulation, and boredom. While the scales used are well-validated, self-report data may be biased due to social desirability or inaccurate self-perception. Future studies could include additional methods, such as behavioral observations, teacher assessments, or physiological measures (e.g., heart rate variability to gauge stress levels), to complement self-report data and provide more objective assessments of these constructs.

Because the study focused on Chinese undergraduate English majors, the generalizability of the findings to other cultural and educational contexts may be limited. Cultural factors—such as attitudes toward learning, teacher-student relationships, and the educational environment—can significantly influence psychological traits and experiences like mindfulness, grit, and boredom. Future research could replicate this study in different cultural settings and among learners of other languages to explore the universality of the findings and identify culturally specific patterns. Moreover, although the qualitative phase provided rich insights into student experiences, it involved a relatively small subset of participants, which may not capture the full diversity of perspectives. Future studies could expand the qualitative component by including a larger and more diverse sample or by employing alternative qualitative methods, such as in-depth interviews or ethnographic observations, to gain a deeper understanding of how students interact with these psychological constructs.

Additionally, the study did not quantitatively model other potential mediators or moderators that might influence the relationships among mindfulness, grit, self-regulation, and L2 boredom. While factors like self-efficacy are related to self-regulation and classroom environment emerged qualitatively, future research could benefit from explicitly incorporating such variables into the statistical models. For example, investigating specific self-efficacy beliefs as distinct predictors or mediators, or testing the potential moderating role of quantitatively measured classroom environment characteristics (like perceived teacher support or task value) could provide valuable nuance. Factors such as language anxiety also warrant inclusion in future quantitative models to provide a more holistic view of the factors contributing to L2 boredom.

Finally, while this study highlights the importance of psychological constructs in mitigating boredom, it does not explore specific interventions or pedagogical strategies to enhance mindfulness, grit, and self-regulation in language learners. Future research could focus on designing and testing targeted interventions—such as mindfulness-based stress reduction programs, grit development workshops, or self-regulation training modules—to assess their impact on reducing L2 boredom and improving engagement and language learning outcomes.

## Data Availability

The data analyzed in this study is subject to the following licenses/restrictions: the datasets generated and analyzed during the current study are available from the corresponding author upon reasonable request. Requests to access these datasets should be directed to JL, jjinglyu@hotmail.com.

## References

[B1] Alamer A. (2021). Grit and language learning: Construct validation of L2-Grit scale and its relation to later vocabulary knowledge. *Educ. Psychol.* 41 544–562. 10.1080/01443410.2020.1867076

[B2] Alamer A. (2022). Having a single language interest autonomously predicts L2 achievement: Addressing the predictive validity of L2 grit. *System* 108:102850. 10.1016/j.system.2022.102850

[B3] Baer R. A. Carmody J. Hunsinger M. (2012). Weekly change in mindfulness and perceived stress in a mindfulness-based stress reduction program. *J. Clin. Psychol.* 68 755–765. 10.1002/jclp.21865 22623334

[B4] Bandura A. (1986). *Social Foundations of thought and Action: A Social Cognitive Theory.* Hoboken, NJ: Prentice-Hall.

[B5] Boekaerts M. Cascallar E. (2006). How far have we moved toward the integration of theory and practice in self-regulation? *Educ. Psychol. Rev.* 18 199–210. 10.1007/s10648-006-9013-4

[B6] Braun V. Clarke V. (2006). Using thematic analysis in psychology. *Qual. Res. Psychol.* 3 77–101. 10.1191/1478088706qp063oa 32100154

[B7] Browne M. W. Cudeck R. (1993). “Alternative ways of assessing model fit,” in *Testing Structural Equation Models*, eds Bollen K. A. Long J. S. (London: Sage), 136–162.

[B8] Carey K. B. Neal D. J. Collins S. E. (2004). A psychometric analysis of the self-regulation questionnaire. *Addict. Behav.* 29 253–260. 10.1016/j.addbeh.2003.08.001 14732414

[B9] Chapman K. E. (2013). *Boredom in the German Foreign Language Classroom.* Madison, WI: The University of Wisconsin-Madison.

[B10] Chen W. Sun P. Yang Z. (2022). Understanding Chinese second language learners’ foreign language learning boredom in online classes: Its conceptual structure and sources. *J. Multiling. Multicult. Dev.* 45 1–17. 10.1080/01434632.2022.2093887

[B11] Cheng L. (2023). Delving into the role of mindfulness on the relationship among creativity, anxiety, and boredom of young EFL learners. *Heliyon* 9:e14820. 10.1016/j.heliyon.2023.e14820 36873160 PMC9975316

[B12] Cheung G. W. Rensvold R. B. (2002). Evaluating goodness-of-fit indexes for testing measurement invariance. *Struct. Equat. Model.* 9 233–255. 10.1207/S15328007SEM0902_5

[B13] Credé M. (2018). What shall we do about grit? A critical review of what we know and what we don’t know. *Educ. Res.* 47 606–611. 10.3102/0013189X18801322 38293548

[B14] Credé M. Tynan M. C. Harms P. D. (2017). Much ado about grit: A meta-analytic synthesis of the grit literature. *J. Pers. Soc. Psychol.* 113 492–511. 10.1037/pspp0000102 27845531

[B15] Creswell J. W. Plano Clark V. L. (2018). *Designing and Conducting Mixed Methods Research*, 3rd Edn. London: Sage.

[B16] Datu J. A. D. Yuen M. Chen G. (2017). Grit and determination: A review of literature with implications for theory and research. *J. Psychol. Counsell. Sch.* 27 168–176. 10.1017/jgc.2016.2

[B17] Demir Y. (2024). L2 grit: A structured approach to preliminary biblio-systematic review. *System* 123:103353. 10.1016/j.system.2024.103353

[B18] Derakhshan A. Fathi J. (2024). Growth mindset, self-efficacy, and self-regulation: A symphony of success in L2 speaking. *System* 123:103320. 10.1016/j.system.2024.103320

[B19] Derakhshan A. Fathi J. (2025). From boredom to buoyancy: examining the impact of perceived teacher support on EFL learners’ resilience and achievement through a serial mediation model. *Innovat. Lang. Learn. Teach.* 1–16. 10.1080/17501229.2025.2487932

[B20] Dörnyei Z. (2009). *The Psychology of Second Language Acquisition.* Oxford: Oxford University Press.

[B21] Dörnyei Z. Henry A. Muir C. (2015). *Motivational Currents in Language Learning: Frameworks for Focused Interventions.* London: Routledge. 10.4324/9781315772714

[B22] Duckworth A. L. Gross J. J. (2014). Self-control and grit: Related but separable determinants of success. *Curr. Dir. Psychol. Sci.* 23 319–325. 10.1177/0963721414541462 26855479 PMC4737958

[B23] Duckworth A. L. Quinn P. D. (2009). Development and validation of the Short Grit Scale (Grit-S). *J. Pers. Assess.* 91 166–174. 10.1080/00223890802634290 19205937

[B24] Duckworth A. L. Peterson C. Matthews M. D. Kelly D. R. (2007). Grit: Perseverance and passion for long-term goals. *J. Pers. Soc. Psychol.* 92 1087–1101. 10.1037/0022-3514.92.6.1087 17547490

[B25] Eastwood J. D. Frischen A. Fenske M. J. Smilek D. (2012). The unengaged mind: Defining boredom in terms of attention. *Perspect. Psychol. Sci.* 7 482–495. 10.1177/1745691612456044 26168505

[B26] Ericsson K. A. Krampe R. T. Tesch-Römer C. (1993). The role of deliberate practice in the acquisition of expert performance. *Psychol. Rev.* 100 363–406. 10.1037/0033-295X.100.3.363

[B27] Eskreis-Winkler L. Shulman E. P. Beal S. A. Duckworth A. L. (2014). The grit effect: Predicting retention in the military, the workplace, school, and marriage. *Front. Psychol.* 5:36. 10.3389/fpsyg.2014.00036 24550863 PMC3910317

[B28] Fathi J. Pawlak M. Kruk M. Naderi M. (2023). Modelling boredom in the EFL context: An investigation of the role of coping self-efficacy, mindfulness, and foreign language enjoyment. *Lang. Teach. Res.* 1–31. 10.1177/13621688231182176

[B29] Fathi J. Pawlak M. Saeedian S. Ghaderi A. (2024). Exploring factors affecting foreign language achievement: The role of growth mindset, self-efficacy, and L2 grit. *Lang. Teach. Res.* 13621688241227603. 10.1177/13621688241227603

[B30] Fornell C. Larcker D. F. (1981). Evaluating structural equation models with unobservable variables and measurement error. *J. Mark. Res.* 18 39–50. 10.1177/002224378101800104

[B31] Ghanizadeh A. Makiabadi H. Navokhi S. A. (2019). Relating EFL university students’ mindfulness and resilience to self-fulfilment and motivation in learning. *Issues Educ. Res.* 29 695–714.

[B32] Greene J. A. (2017). *Self-regulation in Education.* London: Routledge, 10.4324/9781315537450-2

[B33] Hu L. T. Bentler P. M. (1999). Cutoff criteria for fit indexes in covariance structure analysis: Conventional criteria versus new alternatives. *Struct. Equat. Model.* 6 1–55. 10.1080/10705519909540118

[B34] Jin Y. (2024). Motivating students to actively engage in EFL classrooms: Exploring the role of L2 grit and foreign language enjoyment. *Learn. Motiv.* 85:101960. 10.1016/j.lmot.2023.101960

[B35] Kabat-Zinn J. (2003). Mindfulness-based interventions in context: Past, present, and future. *Clin. Psychol.* 10 144–156. 10.1093/clipsy.bpg016

[B36] Kabat-Zinn J. (2015). Mindfulness. *Mindfulness* 6 1481–1483. 10.1007/s12671-015-0456-x

[B37] Khoury B. Lecomte T. Fortin G. Masse M. Therien P. Bouchard V. (2013). Mindfulness-based therapy: A comprehensive meta-analysis. *Clin. Psychol. Rev.* 33 763–771. 10.1016/j.cpr.2013.05.005 23796855

[B38] Kline R. B. (2015). *Principles and Practice of Structural Equation Modeling*, 4th Edn. New York, NY: Guilford Press.

[B39] Krashen S. D. (1982). *Principles and Practice in Second Language Acquisition.* Bergama: Pergamon, 10.1111/j.1467-971X.1982.tb00476.x

[B40] Lamb M. (2017). The motivational dimension of language teaching. *Lang. Teach.* 50 301–346. 10.1017/S0261444817000088

[B41] Lee J. S. (2020). The role of grit and classroom enjoyment in EFL learners’ willingness to communicate. *J. Multiling. Multicult. Dev.* 43 452–468. 10.1080/01434632.2020.1746319

[B42] Lee J. S. (2022). The role of grit and classroom enjoyment in EFL learners’ willingness to communicate. *J. Multiling. Multicult. Dev.* 43 452–468. 10.1080/01434632.2020.1746319

[B43] Li C. (2022). Foreign language learning boredom and enjoyment: The effects of learner variables and teacher variables. *Lang. Teach. Res.* 29 1499–1524. 10.1177/13621688221090324

[B44] Li C. Han Y. (2024). Learner-internal and learner-external factors for boredom amongst Chinese university EFL students. *Appl. Linguist. Rev.* 15 901–926. 10.1515/applirev-2022-0010

[B45] Li C. Wei L. (2023). Anxiety, enjoyment, and boredom in language learning amongst junior secondary students in rural China: How do they contribute to L2 achievement? *Stud. Sec. Lang. Acquisit.* 45 93–108. 10.1017/S0272263122000031

[B46] Li C. Dewaele J. M. Hu Y. (2023). Foreign language learning boredom: Conceptualization and measurement. *Appl. Linguist. Rev.* 14 223–249. 10.1515/applirev-2020-0124

[B47] Liu H. Li X. Guo G. (2025a). Students’ L2 grit, foreign language anxiety and language learning achievement: A latent profile and mediation analysis. *IRAL Int. Rev. Appl. Linguist. Lang. Teach.* 10.1515/iral-2024-0013

[B48] Liu H. Li X. Yan Y. (2025b). Demystifying the predictive role of students’ perceived foreign language teacher support in foreign language anxiety: The mediating role of L2 grit. *J. Multiling. Multicult. Dev.* 46 1095–1108. 10.1080/01434632.2023.2223171

[B49] Mohammad Hosseini H. Derakhshesh A. Fathi J. Mehraein S. (2023). Examining the relationships between mindfulness, grit, academic buoyancy and boredom among EFL learners. *Soc. Psychol. Educ.* 27 1–30. 10.1007/s11218-023-09860-5

[B50] Morgan W. J. Katz J. (2021). Mindfulness meditation and foreign language classroom anxiety: Findings from a randomized control trial. *For. Lang. Ann.* 54 389–409. 10.1111/flan.12509

[B51] Nakamura S. Darasawang P. Reinders H. (2021). The antecedents of boredom in L2 classroom learning. *System* 98:102469. 10.1016/j.system.2021.102469

[B52] Nitta R. Baba K. (2015). “Self-regulation in the evolution of the ideal L2 self: A complex dynamic systems approach to the L2 motivational self system,” in *Motivational Dynamics in Language Learning*, eds Dörnyei Z. Ushioda E. (Leeds: Emerald Publishing Limited), 367–396. 10.21832/9781783092574-023

[B53] Nunnally J. C. Bernstein I. H. (1994). *Psychometric Theory*, 3rd Edn. New York, NY: McGraw-Hill.

[B54] Oxford R. L. (2016). *Teaching and Researching Language Learning Strategies: Self-Regulation in Context.* London: Routledge. 10.4324/9781315719146

[B55] Pawlak M. Kruk M. Zawodniak J. Pasikowski S. (2020). Investigating factors responsible for boredom in English classes: The case of advanced learners. *System* 91:102259. 10.1016/j.system.2020.102259

[B56] Pawlak M. Zarrinabadi N. Kruk M. (2022). Positive and negative emotions, L2 grit and perceived competence as predictors of L2 motivated behaviour. *J. Multiling. Multicult. Dev.* 45 3188–3204. 10.1080/01434632.2022.2091579

[B57] Pekrun R. (2006). Control-value theory of achievement emotions: Assumptions, corollaries, and implications for educational research and practice. *Educ. Psychol. Rev.* 18 315–341. 10.1007/s10648-006-9029-9

[B58] Pekrun R. Goetz T. Daniels L. M. Stupnisky R. H. Perry R. P. (2010). Boredom in achievement settings: Exploring control–value antecedents and performance outcomes of a neglected emotion. *J. Educ. Psychol.* 102 531–549. 10.1037/a0019243

[B59] Pintrich P. R. (2000). “The role of goal orientation in self-regulated learning,” in *Handbook of self-Regulation*, eds Boekaerts M. Pintrich P. R. Zeidner M. (New York, NY: Academic Press), 451–502. 10.1016/B978-012109890-2/50043-3

[B60] Preacher K. J. Hayes A. F. (2008). Asymptotic and resampling strategies for assessing and comparing indirect effects in multiple mediator models. *Behav. Res. Methods* 40 879–891. 10.3758/BRM.40.3.879 18697684

[B61] Rose H. Briggs J. G. Boggs J. A. Sergio L. Ivanova-Slavianskaia N. (2018). A systematic review of language learner strategy research in the face of self-regulation. *System* 72 151–163. 10.1016/j.system.2017.12.002

[B62] Sasaki M. Mizumoto A. Murakami A. (2018). Developmental trajectories in L2 writing strategy use: A self-regulation perspective. *Modern Lang. J.* 102 292–309. 10.1111/modl.12465

[B63] Schunk D. H. Zimmerman B. J. (2023). “Self-regulation in education: Retrospect and prospect,” in *Self-regulation of Learning and Performance*, eds Schunk D. H. Zimmerman B. J. (London: Routledge), 305–314. 10.4324/9780203763353-13

[B64] Seker M. (2016). The use of self-regulation strategies by foreign language learners and its role in language achievement. *Lang. Teach. Res.* 20 600–618. 10.1177/1362168815578550

[B65] Shapiro S. L. Carlson L. E. Astin J. A. Freedman B. (2006). Mechanisms of mindfulness. *J. Clin. Psychol.* 62 373–386. 10.1002/jclp.20237 16385481

[B66] Sharp J. G. Hemmings B. Kay R. Murphy B. Elliott S. (2017). Academic boredom among students in higher education: A mixed-methods exploration of characteristics, contributors and consequences. *J. Further High. Educ.* 41 657–677. 10.1080/0309877X.2016.1159292

[B67] Solhi M. Derakhshan A. Ünsal B. (2023). Associations between EFL students’ L2 grit, boredom coping strategies, and emotion regulation strategies: A structural equation modeling approach. *J. Multiling. Multicult. Dev.* 10.1080/01434632.2023.2175834

[B68] Sun P. P. Zhang J. Zhao X. (2024). Modeling speaking performance in young learners of Chinese as a heritage language: The interplay of L2 grit, motivational intensity, and willingness to communicate. *System* 126:103490. 10.1016/j.system.2024.103490

[B69] Tabachnick B. G. Fidell L. S. (2019). *Using Multivariate Statistics*, 7th Edn. Chennai: Pearson.

[B70] Tashakkori A. Teddlie C. (2010). Putting the human back in “human research methodology”: The researcher in mixed methods research. *J. Mixed Methods Res.* 4 271–277. 10.1177/1558689810382532

[B71] Teimouri Y. Plonsky L. Tabandeh F. (2022). L2 grit: Passion and perseverance for second-language learning. *Lang. Teach. Res.* 26 893–918. 10.1177/1362168820921895

[B72] Teimouri Y. Sudina E. Plonsky L. (2021). On domain-specific conceptualization and measurement of grit in L2 learning. *J. Psychol. Lang. Learn.* 3 156–165. 10.52598/jpll/3/2/4

[B73] Teng L. S. Zhang L. J. (2022). Can self-regulation be transferred to second/foreign language learning and teaching? Current status, controversies, and future directions. *Appl. Linguist.* 43 587–595. 10.1093/applin/amab032

[B74] Torres G. (2013). Empowering the language learner: Language learning strategy training and self-regulation in an EFL classroom. *J. Int. Educ. Res.* 9 267–276. 10.19030/jier.v9i3.7876

[B75] Tsuda A. Nakata Y. (2013). Exploring self-regulation in language learning: A study of Japanese high school EFL students. *Innovat. Lang. Learn. Teach.* 7 72–88. 10.1080/17501229.2012.686500

[B76] Tze V. M. Daniels L. M. Klassen R. M. (2016). Evaluating the relationship between boredom and academic outcomes: A meta-analysis. *Educ. Psychol. Rev.* 28 119–144. 10.1007/s10648-015-9301-y

[B77] Wei R. Liu H. Wang S. (2020). Exploring L2 grit in the Chinese EFL context. *System* 93:102295. 10.1016/j.system.2020.102295

[B78] West S. G. (1995). “Structural equation models with nonnormal variables: Problems and remedies,” in *Structural Equation Modeling: Concepts, Issues and Applications*, ed. Hoyle R. H. (London: Sage).

[B79] Wu J. Zhao Q. (2023). The contribution of mindfulness in the association between L2 learners’ engagement and burnout. *Heliyon* 9:e14784. 10.1016/j.heliyon.2023.e14784 38027673 PMC10663844

[B80] Zeilhofer L. (2023). Mindfulness in the foreign language classroom: Influence on academic achievement and awareness. *Lang. Teach. Res.* 27 96–114. 10.1177/1362168820934624

[B81] Zeilhofer L. Sasao Y. (2022). Mindful language learning: The effects of college students’ mindfulness on short-term vocabulary retention. *System* 110:102909. 10.1016/j.system.2022.102909

[B82] Zhang L. J. Saeedian A. Fathi J. (2022). Testing a model of growth mindset, ideal L2 self, boredom, and WTC in an EFL context. *J. Multiling. Multicult. Dev.* 45 3450–3465. 10.1080/01434632.2022.2100893

[B83] Zhao X. Wang D. (2023). Grit, emotions, and their effects on ethnic minority students’ English language learning achievements: A structural equation modelling analysis. *System* 113:102979. 10.1016/j.system.2023.102979

[B84] Zhao X. Wang D. (2024). Domain-specific L2 grit, anxiety, boredom, and enjoyment in online Chinese learning. *Asia Pac. Educ. Res.* 33 783–794. 10.1007/s40299-023-00777-3

[B85] Zhao X. Sun P. P. Gong M. (2024). The merit of grit and emotions in L2 Chinese online language achievement: A case of Arabian students. *Int. J. Multiling.* 21 1653–1679. 10.1080/14790718.2023.2202403

[B86] Zimmerman B. J. (2000). “Attaining self-regulation: A social cognitive perspective,” in *Handbook of self-regulation*, eds Vohs K. D. Baumeister R. F. (New York, NY: Academic Press), 13–39. 10.1016/B978-012109890-2/50031-7

[B87] Zimmerman B. J. (2002). Becoming a self-regulated learner: An overview. *Theory Pract.* 41 64–70. 10.1207/s15430421tip4102_2

[B88] Zimmerman B. J. Kitsantas A. (2014). Comparing students’ self-discipline and self-regulation measures and their prediction of academic achievement. *Contemp. Educ. Psychol.* 39 145–155. 10.1016/j.cedpsych.2014.03.004

